# Dietary intervention reverses molecular markers of hepatocellular senescence in the GAN diet-induced obese and biopsy-confirmed mouse model of NASH

**DOI:** 10.1186/s12876-024-03141-x

**Published:** 2024-02-02

**Authors:** Mathias Flensted-Jensen, Denise Oró, Emma A. Rørbeck, Chen Zhang, Martin Rønn Madsen, Andreas Nygaard Madsen, Jenny Norlin, Michael Feigh, Steen Larsen, Henrik H. Hansen

**Affiliations:** 1 Gubra, Hørsholm Kongevej 11B, 2970 Hørsholm, Denmark; 2 Xlab, Department of Biomedical Sciences, University of Copenhagen, Blegdamsvej 3, 2200 Copenhagen, Denmark; 3 Liver Disease Research, Novo Nordisk A/S, Måløv, Denmark; 4 Clinical Research Centre, Medical University of Bialystok, Bialystok, Poland; 5 Present address: Novo Nordisk A/S, Beijing, China

**Keywords:** Non-alcoholic steatohepatitis, Fibrosis, Animal model, Hepatocellular senescence, Dietary intervention, Mitochondrial respiration

## Abstract

**Background:**

Hepatocellular senescence may be a causal factor in the development and progression of non-alcoholic steatohepatitis (NASH). The most effective currently available treatment for NASH is lifestyle intervention, including dietary modification. This study aimed to evaluate the effects of dietary intervention on hallmarks of NASH and molecular signatures of hepatocellular senescence in the Gubra-Amylin NASH (GAN) diet-induced obese (DIO) and biopsy-confirmed mouse model of NASH.

**Methods:**

GAN DIO-NASH mice with liver biopsy-confirmed NASH and fibrosis received dietary intervention by switching to chow feeding (chow reversal) for 8, 16 or 24 weeks. Untreated GAN DIO-NASH mice and chow-fed C57BL/6J mice served as controls. Pre-to-post liver biopsy histology was performed for within-subject evaluation of NAFLD Activity Score and fibrosis stage. Terminal endpoints included blood/liver biochemistry, quantitative liver histology, mitochondrial respiration and RNA sequencing.

**Results:**

Chow-reversal promoted substantial benefits on metabolic outcomes and liver histology, as demonstrated by robust weight loss, complete resolution of hepatomegaly, hypercholesterolemia, elevated transaminase levels and hepatic steatosis in addition to attenuation of inflammatory markers. Notably, all DIO-NASH mice demonstrated ≥ 2 point significant improvement in NAFLD Activity Score following dietary intervention. While not improving fibrosis stage, chow-reversal reduced quantitative fibrosis markers (PSR, collagen 1a1, α-SMA), concurrent with improved liver mitochondrial respiration, complete reversal of p21 overexpression, lowered γ-H2AX levels and widespread suppression of gene expression markers of hepatocellular senescence.

**Conclusions:**

Dietary intervention (chow reversal) substantially improves metabolic, biochemical and histological hallmarks of NASH and fibrosis in GAN DIO-NASH mice. These benefits were reflected by progressive clearance of senescent hepatocellular cells, making the model suitable for profiling potential senotherapeutics in preclinical drug discovery for NASH.

## Introduction

Non-alcoholic fatty liver disease (NAFLD) has become the most prevalent liver disease worldwide, affecting approximately 25% of the world’s population [1]. NAFLD represents a wide spectrum of liver diseases, ranging from benign steatosis to the most advanced and severe form, non-alcoholic steatohepatitis (NASH) [2]. The strongest risk factors for NASH include obesity, dyslipidemia and type 2 diabetes [3, 4]. Given the epidemic proportions of these metabolic diseases, the disease burden of NASH is expected to grow significantly in the future [5]. NASH is characterized by the presence of steatosis, lobular inflammation and hepatocellular ballooning [6], and is strongly associated with the development of liver fibrosis which is the major driver for cardiovascular co-morbidity, malignancy and mortality in NASH [7].

There is an increasing appreciation that cellular senescence, a state of irreversible cell cycle arrest, plays an important role in the pathogenesis of metabolic disease complications, including NASH [8–10]. Cellular senescence can be induced by a variety of stressors such as telomere dysfunction as well as genotoxic and oxidative stress [11]. Notably, cellular senescence is characterized by expression and secretion of distinct pro-inflammatory factors, termed the senescence-associated secretory phenotype (SASP), which can have deleterious effects on the tissue microenvironment and contribute to hepatic stellate cell activation and fibrotic injury [12, 13]. Accordingly, emerging preclinical and clinical evidence suggests that accelerated hepatocellular senescence is an important factor in the sequelae of NAFLD/NASH [8–10, 14]. NAFLD/NASH patients show increases in established hepatocellular senescence markers, including cell cycle arrest (p21) and phosphorylation of histone H2AX (γ-H2AX), which correlate with the severity of the disease [14–16]. A series of preclinical studies have pointed to hepatocyte-specific senescence playing a causal role in liver fat accumulation by interfering with β-oxidative fatty acid disposal [14, 17]. In support, clearance of senescent hepatocytes by senolytic drugs reduces steatosis in high-fat diet-induced obese (DIO) mouse models of NAFLD/NASH [14].

It has become increasingly evident that cellular senescence is associated with mitochondrial dysfunction. Given that mitochondria are critically involved in cell survival and death [18], and the main source of reactive oxygen species (ROS) [19], mitochondrial dysfunction can trigger cellular senescence programs [20–22]. Conversely, cellular senescence can also lead to mitochondrial dysfunction. Accordingly, senescent cells have been reported to accumulate mitochondria with lower membrane potential and respiration rate as well as increased ROS production [14, 22]. It has therefore been proposed that deficient mitochondrial homeostasis underlies hepatocellular senescence in NASH [14, 23].

Lifestyle intervention remains first-line treatment for NASH as no evidence-based drug treatments have been approved for the disease [24]. The lack of effective treatments has been attributed to the lack of understanding of the many different molecular pathways that contribute to the onset and progression of NASH [25]. As a consequence, there is a critical need for exploring novel molecular mechanisms that could be potentially targeted for reducing liver injury in NASH patients. Many rodent models of NASH lack sufficient validation with respect to disease progression and treatment intervention which limits their use in preclinical drug discovery. Given the therapeutic potential of reducing hepatocellular senescence in NASH, we profiled the GAN diet-induced obese (DIO) mouse model of NASH (GAN DIO-NASH), an established translational mouse model of biopsy-confirmed NASH and fibrosis [26, 27], for molecular and mitochondrial markers of hepatocellular senescence. In addition, dietary intervention was probed for efficacy on hepatocellular senescence markers in the model.

## Methods

### Animals

C57BL/6J mice (5–6 weeks old, *n* = 106) were from Janvier Labs (Le Genest Saint Isle, France) and housed in a controlled environment (12 h light/dark cycle, lights on at 3 a.m., 21 ± 2°C, humidity 50 ± 10%). Each animal was identified by an implantable subcutaneous microchip (PetID Microchip, E-vet, Haderslev, Denmark). Mice had ad libitum access to tap water and chow (3.22 kcal/g, Altromin 1324; Brogaarden, Hoersholm, Denmark) or Gubra Amylin NASH diet ([GAN diet, 4.49 kcal/g, 40 kcal-% fat] of these 46% saturated fatty acids by weight, 22% fructose, 10% sucrose, 2% cholesterol; D09100310, Research Diets). Mice were fed chow or the GAN diet for up to 58 weeks. Animals were terminated by cardiac puncture under isoflurane anesthesia.

### Baseline liver biopsy

Animals underwent liver biopsy before treatment intervention, as described in detail previously [28]. Mice were anesthetized with isoflurane, a midline abdominal incision was made to expose the left lateral lobe, and a cone-shaped biopsy of ~ 50 mg liver tissue was collected. Cut surfaces were electro-coagulated using an electrosurgical unit. Thereafter, the liver was returned to the abdominal cavity, the abdominal wall was sutured, and the skin was stapled. Animals received 5 mg/kg carprofen prior to surgery and on postoperative days 1 and 2. Animals were single-housed after the procedure and allowed to recover for 4 weeks prior to treatment start.

### Dietary intervention

**Fig. 1:**
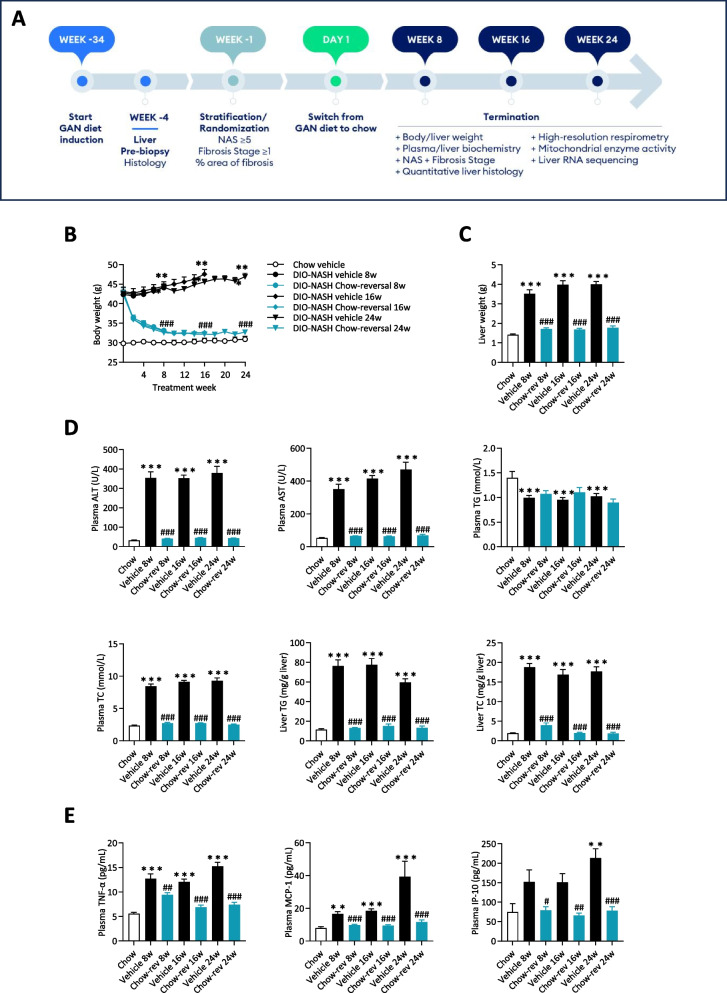
Dietary intervention improves metabolic markers in GAN DIO-NASH mice. Mice were fed the GAN diet for 34 weeks before switching to chow feeding (chow-reversal) for 8, 16 or 24 weeks (*n* = 14-16 per group). Chow-fed C57BL/6J mice (Chow vehicle) served as normal controls (*n* = 10). All mice received vehicle (SC, QD, 5 ml/kg) during the intervention phase of the study. **A** Study outline. **B **Body weight profile and terminal body weight. **C** Terminal liver weight. **D** Plasma and liver biochemistry. Plasma ALT; plasma AST; plasma triglycerides; plasma total cholesterol; liver triglycerides; liver total cholesterol. **E** Plasma cytokine levels of TNF-α, MCP-1 and IP-10. Mean ± SEM. **p < 0.01, ***p < 0.001 compared to chow vehicle; ^#^*p* < 0.05, ^##^*p* < 0.01, ^###^*p* < 0.001 compared to corresponding GAN DIO-NASH vehicle control group. Dunnett’s test one-factor linear model

See study outline in Fig. 1A. Animals were fed the GAN diet (vehicle and chow reversal groups), or chow (chow vehicle) for 34 weeks. Only DIO-NASH mice with biopsy-confirmed severe steatosis (score 3) and fibrosis (≥ stage F1) were included in the study, evaluated using standard clinical biopsy histopathological scoring criteria [29]. Mice were randomized and stratified to treatment based on baseline mean steatosis, fibrosis score and %-area of fibrosis (picro-Sirius Red, PSR). DIO-NASH mice were administered saline vehicle (SC, QD, 5 ml/kg) with or without switching from the GAN diet to chow (Chow reversal) for 8, 16 or 24 weeks (*n* = 14–16 per group). Chow-fed mice (*n* = 9) dosed with vehicle for 24 weeks served as normal controls. Body weight was measured daily.

### Plasma and liver biochemistry

Four-hour fasted terminal blood was sampled from the tail vein, kept on ice, and centrifuged (5 min, 4°C; 6,000 g) to generate EDTA-stabilized plasma. Plasma alanine aminotransferase (ALT), aspartate aminotransferase, triglycerides (TG), and total cholesterol (TC), as well as liver TG and TC was determined, as described previously [30]. For plasma cytokine analysis (TNF-α, IP10 and MCP-1), a customized U-plex multiplex assay was applied to plasma samples according to the manufacturer's protocol, utilizing recommended sample dilutions and standard curve concentrations (Meso Scale Diagnostics, Rockville, MD).

### Liver histology

Baseline liver biopsy and terminal samples (both from the left lateral lobe) were fixed overnight in 4% paraformaldehyde. Liver tissue was paraffin-embedded and sectioned (3 µm thickness). Sections were stained with hematoxylin-eosin (HE), PSR (Sigma-Aldrich, Brøndby, Denmark), anti-galectin-3 (cat. 125402; Biolegend, San Diego, CA), alpha-smooth muscle actin (α-SMA, cat. ab124964; Abcam, Cambridge, UK), or anti-type I collagen (Col1a1, cat. 1310-01; Southern Biotech, Birmingham, AL), using standard procedures [28, 30]. For detection of hepatocellular senescence, liver sections were stained with anti-p21 antibody (1:500, cat. ab188224, Abcam, Cambridge, UK) and anti-γ-H2AX (1:1000, cat. ab26350, Abcam, Cambridge, UK), incubated with secondary antibodies Alexa-Fluor-647 (1:100, cat. 711606152, Jackson ImmunoResearch, Cambridge, UK) and Alexa-Fluor-555 (1:2000, cat. ab150114, Abcam, Cambridge, UK) and counterstained with DAPI (1:1000, cat. D9542, Sigma-Aldrich, Brøndby, Denmark) to visualize nuclei. An automated deep learning-based digital imaging analysis pipeline [Gubra Histopathological Objective Scoring Technology, GHOST [31]] was applied for automated histopathological scoring using the NASH Clinical Research Network (CRN) scoring system [29]. In addition, GHOST was applied to histopathological scoring variables for unbiased quantification of whole-section number of lipid-laden hepatocytes (expressed relative to total number of hepatocytes), number of inflammatory foci (foci per mm^2^), and proportionate (%) area of perisinusoidal and periportal fibrosis [31]. Quantitative histomorphometry was performed using a digital imaging software (Visiomorph; Visiopharm, Hørsholm, Denmark) for the determination of whole-section liver fat (HE-staining), fibrosis (PSR, Col1a1), inflammation (galectin-3), and hepatic stellate cell (HSC) activation (α-SMA), expressed relative (%) to total sectional area. p21, ϒ-H2AX and p21 + ϒ-H2AX stained cells were counted and expressed as number of immunopositive cells per 10,000 DAPI-stained cell nuclei (34).

### High-resolution respirometry

Fresh liver samples were dissected and washed in MiR05 buffer (EGTA 0.5 mM, MgCl_2_.6H_2_O 3 mM, K‐lactobionate 60 mM, taurine 20 mM, KH_2_PO_4_ 10 mM, HEPES 20 mM, sucrose 110 mM, BSA 1 g/L at pH 7.1), before being added to an oxygraph (Oxygraph-2k; Oroboros, Innsbruck, Austria) for measurements of mitochondrial respiratory capacity (MRC). All measurements were done in duplicate at 37 °C in a hyperoxygenated chamber (450 nmol O_2_/ml). Pyruvate (5 mM), malate (2 mM) and glutamate (10 mM), and ADP (5 mM) in combination with magnesium chloride (3 mM), was added to assess MRC when only complex I of the electron transport system (ETS) was active (complex I respiration). Next, cytochrome C (0.01 mM) was added to control for outer mitochondrial membrane integrity. We saw no change in respiration after the addition of cytochrome C, indicating that the outer mitochondrial membrane maintained its integrity (data not shown). Thereafter, succinate (10 mM) was added to assess mitochondrial respiratory chain (MRC) activity when complex I and II, as well as other complexes of the ETC, were active (complex I + II respiration). Finally, carbonyl cyanide p-trifluoro-methoxyphenyl hydrazone (FCCP) was added in steps of 0.5 µl (0.25 µM) until there was no further increase in respiration to assess maximally uncoupled respiration (Respiration-ETS), as a measurement of the capacity of ETS. Respiratory capacity was normalized to liver tissue wet weight and expressed as pmol O_2_/sec/mg tissue.

### Mitochondrial enzyme activity

Analysis of enzyme activities of beta-hydroxyacyl-CoA dehydrogenase (β-HAD) and citrate synthase was performed using approximately 2 mg of liver tissue. Homogenization was conducted in 600 μl 0.3M K_2_HPO_4_, 0.05% bovine serum albumin at pH 7.7 for 2 min using a Tissuelyser (Qiagen, Venlo, Limburg, the Netherlands). Six microliter of 10% Triton was added, whereupon the liver samples were left on ice for 15 min and then stored at − 80°C for later analyses by spectrophotometry (Cobas 6000 c501, Roche, Glostrup, Denmark).

### RNA sequencing

Liver transcriptomics were performed using RNA sequencing (RNAseq) on RNA extracts from terminal liver samples (15 mg fresh tissue), as described in detail elsewhere [27]. RNA sequence libraries were prepared using the NEBNext Ultra II Directional RNA Library Prep Kit for Illumina (New England Biolabs, Ipswich, USA) and sequenced on the NextSeq 500 (Illumina) with NextSeq 500/550 High Output Kit V2 (75 CYS, Illumina, San Diego, USA). Reads were aligned to the GRCm38 version 96 Ensembl Mus musculus genome using STAR version 2.7.0f with default parameters. Downstream RNAseq data analysis was performed using the statistical software R. The R-package DESeq2 was used for differential gene expression analysis, and p values were corrected for multiple testing using the Benjamini–Hochberg method (5% false discovery rate, *p* < 0.05).

### Statistics

Except from deep learning-based image analysis and RNA sequencing, data were analyzed using GraphPad Prism version 9.5.1 software (GraphPad, La Jolla, CA). All results are indicated as mean ± standard error of mean (S.E.M.). A one-sided Fisher’s exact test with Bonferroni correction was used for within-subject comparison of histopathological scores before and after treatment intervention. A Dunnett’s test one- or two-factor linear model with interaction was used for all other statistical analyses. A *p*-value less than 0.05 was considered statistically significant.

## Results

### Dietary intervention improves hallmarks of NASH and fibrosis in GAN DIO-NASH mice

**Fig. 2:**
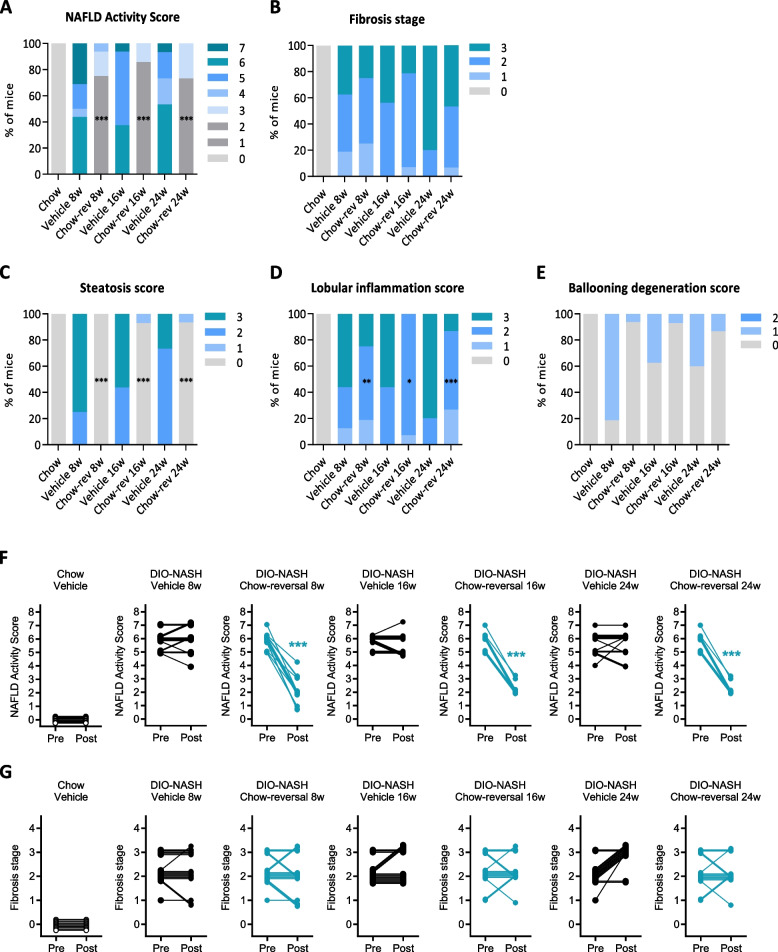
Dietary intervention improves NAFLD Activity Score but not fibrosis stage in GAN DIO-NASH mice. **A** NAFLD Activity Score (NAS). **B** Fibrosis stage. **C** Steatosis score. **D** Lobular inflammation score. **E** ballooning degeneration score. *p < 0.05, **p < 0.01, ***p < 0.001 compared to corresponding vehicle control group. One-sided Fisher’s exact test with Bonferroni correction. Comparison of individual pre-post liver biopsy histopathological scores, including (**F**) NAFLD Activity Score (NAS; composite of steatosis, lobular inflammation and hepatocyte ballooning degeneration scores) and (**G**) Fibrosis stage. ***p < 0.001 vs. corresponding DIO-NASH vehicle control group (one-sided Fisher’s exact test with Bonferroni correction)

**Fig. 3:**
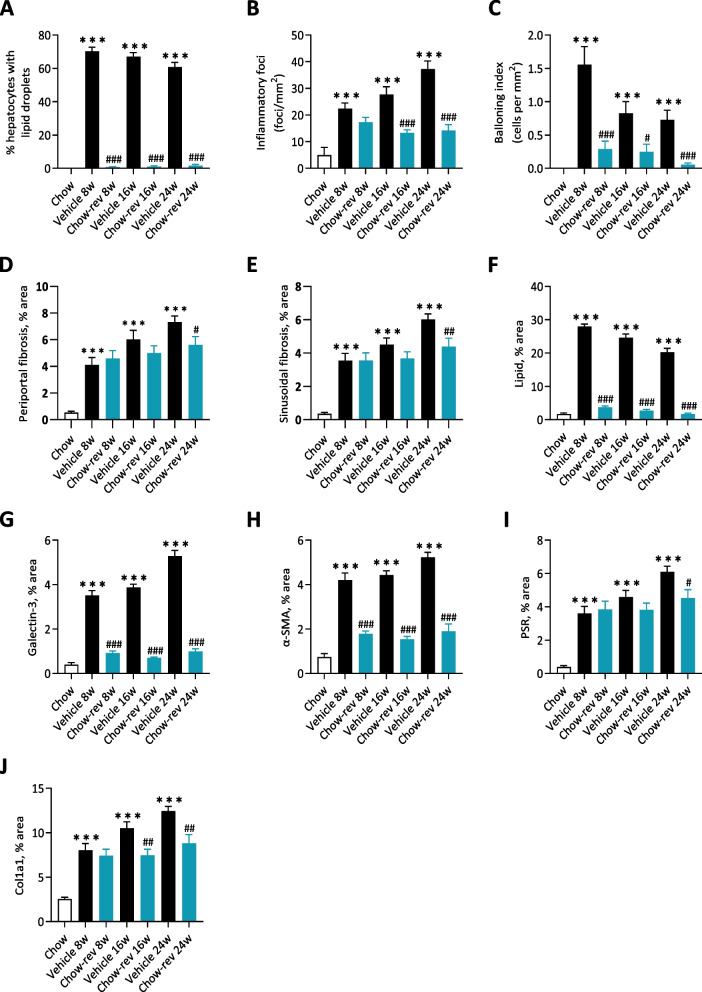
Dietary intervention improves quantitative histological markers of NASH and fibrosis in GAN DIO-NASH mice. **A-E** Automated GHOST-based histomorphometrics on histopathological scoring-derived variables, including (**A**) % hepatocytes with lipid droplets; (**B**) Number of inflammatory foci; (**C**) Ballooning index; (**D**) % area of periportal fibrosis; (**E**) % area of sinusoidal fibrosis. **F-J** Conventional quantitative image analysis (% area) of (**F**) Lipids; (**G**) Galectin-3.; (**H**) α-smooth muscle actin (α-SMA); (**I**) Fibrosis (PSR); (**J**) Fibrosis (collagen-1a1); ***p < 0.001 (compared to chow vehicle); ^#^*p* < 0.05, ^##^*p* < 0.01, ^###^*p* < 0.001 compared to corresponding GAN DIO-NASH mouse vehicle control group. Dunnett’s test one-factor linear model

**Fig. 4:**
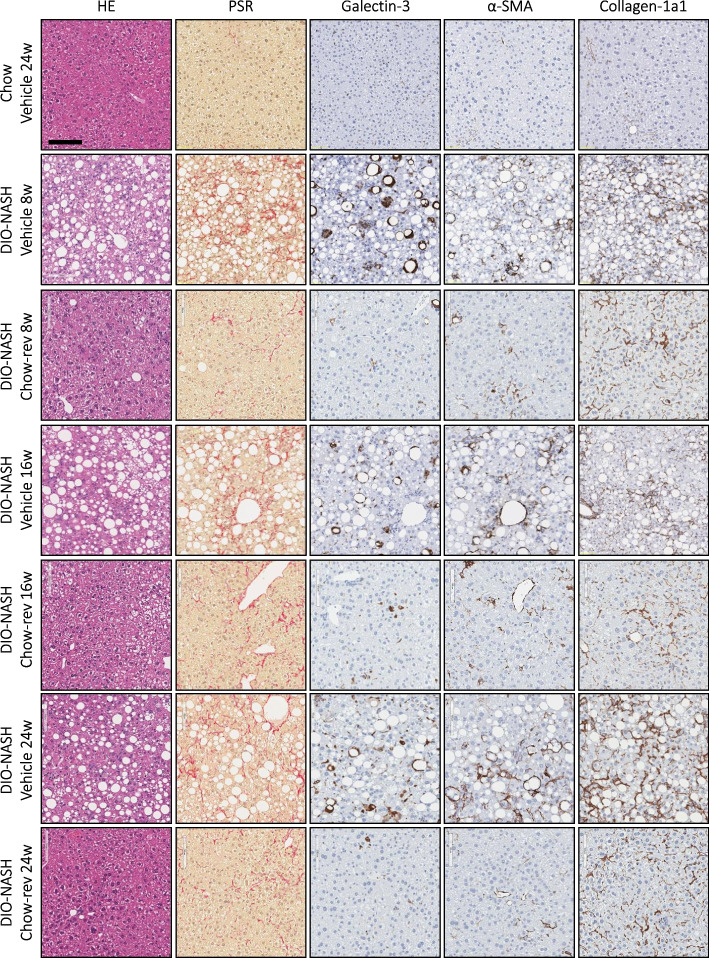
Dietary intervention improves quantitative histopathological hallmarks of NASH and fibrosis in GAN DIO-NASH mice. Representative photomicrographs of HE, PSR, galectin-3, α-SMA and Col1a1 stainings following dietary intervention (chow reversal, Chow rev) for 8, 16 and 24 weeks in GAN DIO-NASH mice (scale bar, 100 µm). Mice were fed the GAN diet for 34 weeks before switching to chow feeding (chow-reversal) once daily for 8, 16 or 24 weeks, or continued on the GAN diet for 8, 16 or 24 weeks (*n* = 14-16 per group). Chow-fed C57BL/6J mice served controls (*n* = 10). All mice received vehicle (SC, QD, 5 ml/kg) during the intervention phase of the study

**Fig. 5:**
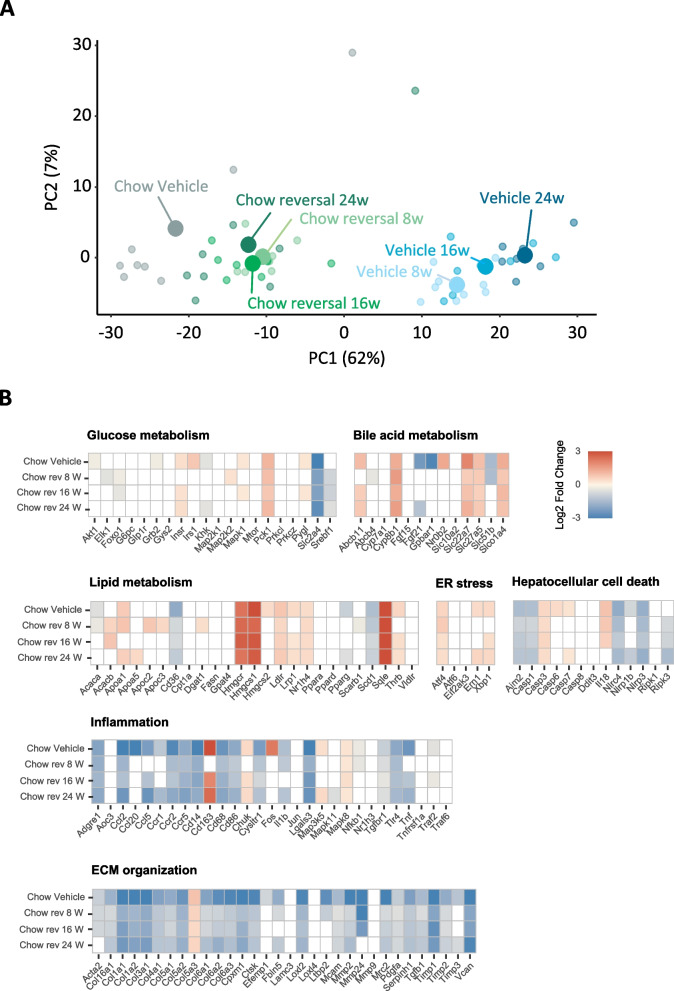
Dietary intervention improves NASH- and fibrosis associated hepatic transcriptome profile in GAN DIO-NASH mice. **A** Principal component analysis (PCA) of samples based on top 500 most variable gene expression levels. **B** Heatmaps illustrating changes in NASH and fibrosis-associated candidate gene expression after 8-24 weeks of chow feeding (Chow reversal, Chow rev) compared to corresponding GAN DIO-NASH vehicle control group. Color gradients in heatmaps indicate significantly upregulated (red color) or downregulated (blue color) gene expression compared to corresponding vehicle-dosed GAN DIO-NASH mouse group (log2-fold change, false discovery rate *p* < 0.05). Unregulated genes are indicated by no color fill (white color)

Dietary intervention reduced body weight (8 weeks, 21.6 ± 1.3%, *p* < 0.001; 16 weeks, 24.5 ± 1.3%, *p* < 0.001; 24 weeks, 22.6 ± 2.1%, *p* < 0.001, Fig. 1B) and hepatomegaly (Fig. 1C), with concurrent complete normalization of plasma ALT, AST as well as liver triglyceride and plasma/liver total cholesterol levels (Fig. 1D). Also, plasma levels of TNF-α, MCP-1 and IP10 were normalized following 8, 16 and 24 weeks of chow reversal (Fig. 1E). Dietary intervention led to a ≥ 2-point improvement in NAFLD Activity Score (NAS, Fig. 2A, F), largely ascribed to improved steatosis and lobular inflammation scores (Fig. 2C, and D). Hepatocyte ballooning was marginal and unaffected by chow reversal (Fig. 2E). Fibrosis stage was not improved after up to 24 weeks of chow reversal in GAN DIO-NASH mice (Fig. 2B, G). GHOST deep learning-based histomorphometrics on histopathological scoring variables supported complete reversal of hepatic lipid accumulation (% of hepatocytes with lipid droplets, %-area of lipids) and markedly reduced inflammation (foci/mm^2^, %-area of galectin-3) after 8 weeks of chow reversal in GAN DIO-NASH mice, with no further improvements in these endpoints after prolonged chow reversal (Figs. 3 and 4). Quantitative fibrosis histological endpoints based on PSR staining analysis (%-area of periportal fibrosis, sinusoidal fibrosis and total fibrosis area) were consistently reduced only after 24 weeks of chow reversal (Figs. 3D, E, I and 4). Significantly reduced proportionate area of collagen-1a1 staining was observed after both 16 and 24 weeks of chow reversal (Figs. 3J and 4). Reduced pro-fibrogenic activity was supported by markedly lowered α-SMA levels after chow reversal, irrespectively of the dietary intervention period applied (Figs. 3H and 4). Changes in hepatic gene expression signatures were highly consistent and relatively similar following 8, 16 and 24 weeks of intervention (Fig. 5A). As for histological endpoints, dietary intervention resulted in robust suppression of gene expression markers of inflammation and fibrogenesis (Fig. 5B).

### Dietary intervention improves hepatic mitochondrial function in GAN DIO-NASH mice

**Fig. 6:**
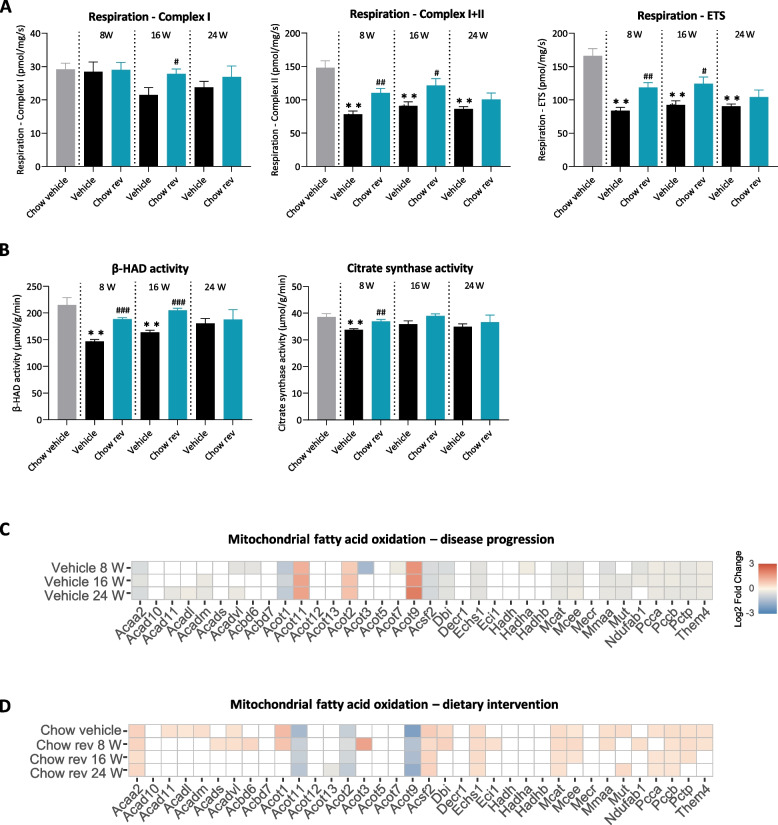
Dietary intervention improves markers of hepatic mitochondrial function in GAN DIO-NASH mice. **A** Maximal liver mitochondrial respiratory capacity assessed via high-resolution respirometry (O_2_ flux/mg tissue/second). Maximal respiration after the addition of substrates stimulating complex I of the electron transport system (ETS; Respiration-Complex I). Maximal respiration after the addition of substrates stimulating both complex I and II of the electron transport chain (Respiration-Complex I + II). Maximal uncoupled respiration after the addition of uncoupling agent FCCP (Respiration-ETS). **B** Spectrophotometrically determined mitochondrial enzyme activity (µmol/g tissue/minute) of enzymes Beta-hydroxyacyl-CoA dehydrogenase (β-HAD) and citrate synthase. Mean ± SEM. **p < 0.01 compared to Chow vehicle; ^#^*p* < 0.05, ^##^*p* < 0.01, ^###^*p* < 0.001 compared to corresponding vehicle-dosed GAN DIO-NASH mouse group. Dunnett’s test one-factor linear model. **C-D** RNA sequencing analysis. **C** Heatmap illustrating changes in expression of genes related to mitochondrial fatty acid oxidation after 8-24 weeks in vehicle-dosed GAN DIO-NASH mice receiving chow reversal as compared to chow controls. **D** Heatmap illustrating changes in expression of genes related to mitochondrial fatty acid oxidation after 8-24 weeks of chow reversal as compared to corresponding GAN DIO-NASH vehicle control group. Color gradients in heatmaps indicate significantly upregulated (red color) or downregulated (blue color) gene expression (log2-fold change, false discovery rate *p* < 0.05). Unregulated genes are indicated without color fill (white color)

While there was no significant reduction in complex I MRC (Respiration-Complex I), maximally stimulated MRC (Respiration Complex I-II) was significantly lowered in vehicle-dosed GAN DIO-NASH mice (8 weeks, 78 ± 4.8 pmol/mg/s; 16 weeks, 91 ± 6.0 pmol/mg/s, 24 weeks, 86 ± 3.5 pmol/mg/s, all *p* < 0.001) compared chow-fed controls (148 ± 10 pmol/mg/s), see Fig 6A.  Also, deficient mitochondrial respiration was supported by lowered maximal uncoupled respiration (Respiration-ETS) in all vehicle-dosed GAN DIO-NASH mouse groups (8 weeks, 84 ± 4.8 pmol/mg/s; 16 weeks; 92 ± 6.3 pmol/mg/s; 24 weeks, 90 ± 3.2 pmol/mg/s; all *p* < 0.001) compared to the corresponding level in chow-fed controls (166 ± 11 pmol/mg/s), see Fig. 6A. Compared to corresponding GAN DIO-NASH controls, dietary intervention resulted in partial normalization of mitochondrial respiratory capacity (Respiration Complex I-II) following 8 weeks (110 ± 7.0, *p* < 0.01) and 16 weeks (122 ± 10, *p* < 0.05), but not 24 weeks (101 ± 9.6, *p* > 0.05), see Fig. 6A. Similarly, maximal coupled respiration (Respiration-ETS) was significantly increased after 8 weeks (119 ± 7.5 vs. 84 ± 4.6 pmol/mg/s, *p* < 0.01) and 16 weeks (124 ± 10 vs. 92 ± 6.3 pmol/mg/s, *p* < 0.05), but not 24 weeks (104 ± 10 vs. 90 ± 3.2 pmol/mg/s, *p* > 0.05) of dietary intervention compared to GAN DIO-NASH controls (Fig. 6A). Impaired hepatic fatty acid oxidative capacity in GAN DIO-NASH mice was suggested by reduced mitochondrial β-HAD activity in the 8- and 16-week GAN DIO-NASH control groups (8 weeks, 147 ± 3.2 µmol/g/min, *p* < 0.001; 16 weeks, 164 ± 4.2 µmol/g/min, *p* < 0.001), but not 24-week control group (181 ± 9.1 µmol/g/min, *p* > 0.05) as compared to chow controls (215 ± 13 µmol/g/min, *p* > 0.05). Dietary intervention for 8 and 16 weeks significantly improved β-HAD activity in GAN DIO-NASH mice (8 weeks, 188 ± 3.0 µmol/g/min, *p* < 0.001; 16 weeks, 205 ± 3.8 µmol/g/min, *p* < 0.001; 24 weeks, 188 ± 18.5 µmol/g/min, *p* > 0.05), see Fig. 6B. Citrate synthase activity, a surrogate marker for mitochondrial density, was slightly but significantly lowered in the 8-week GAN DIO-NASH control group with no significant changes observed in the 16- and 24-week groups (Fig. 6B). Consistent with reduced mitochondrial β-HAD activity, a subset of genes related to hepatic mitochondrial fatty acid oxidation were significantly downregulated in all GAN DIO-NASH control group (Fig. 6C), which was partially reversed by dietary intervention (Fig. 6D).

### Dietary intervention reduces molecular markers of hepatocellular senescence in GAN DIO-NASH mice

**Fig. 7:**
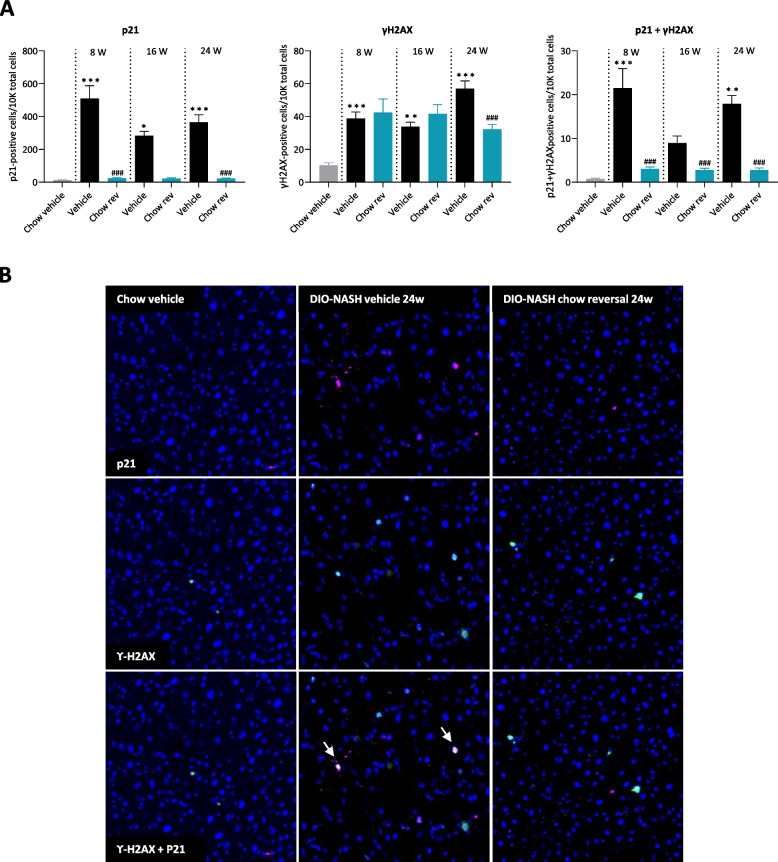
Dietary intervention reduces histological markers of hepatocellular senescence in GAN DIO-NASH mice. **A** Liver histomorphometric quantification of p21, ϒ-H2AX and p21 + ϒ-H2AX positive cells (number of cells per 10,000 DAPI-stained cells). Mean ± SEM. *p < 0.05, **p < 0.01, ***p < 0.001 (compared to Chow Vehicle); ^###^*p* < 0.001 compared to corresponding GAN DIO-NASH vehicle control group. Dunnett’s test one-factor linear model. **B** Representative images of p21, ϒ-H2AX and DAPI co-staining in normal mice (Chow vehicle), vehicle-dosed GAN DIO-NASH mice (DIO-NASH vehicle 24w) and GAN DIO-NASH mice receiving dietary intervention for 24 weeks (DIO-NASH chow reversal 24w). Arrows indicate cells co-expressing p21 and ϒ-H2AX

Chow-fed control mice showed very low levels of hepatic cells positive for p21 (12 ± 2 cells/10,000 cells), ϒ-H2AX (10.3 ± 1.5 cells/10,000 cells), and p21 + ϒ-H2AX co-expression (0.7 ± 0.2 cells/10,000 cells). In GAN DIO-NASH mice, p21-positive cells were approximately tenfold more frequent than ϒ-H2AX positive cells (Fig. 7A, B). The proportion of p21-positive cells was substantially increased (up to 40-fold) in the GAN DIO-NASH control groups as compared to chow-fed controls. In comparison, the number of ϒ-H2AX immunopositive cells were up to fivefold higher in GAN DIO-NASH mice. Correspondingly, the proportion of p21 + ϒ-H2AX co-labelled cells were also significantly elevated in in GAN DIO-NASH mice (Fig. 7A, B). Notably, normalization of p21 expression was observed after 8 weeks of chow reversal and was maintained at baseline levels after extended dietary intervention. The number of ϒ-H2AX positive cells was significantly reduced only after 24 weeks of dietary intervention. Dietary intervention also reduced the number of p21 + ϒ-H2AX co-expressing cells (Fig. 7A, B).

**Fig. 8:**
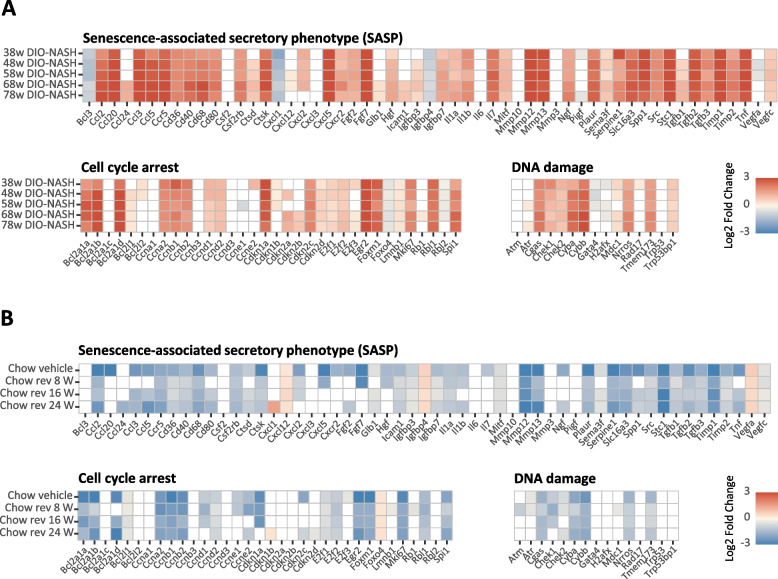
Dietary intervention suppresses gene expression markers of hepatocellular senescence in GAN DIO-NASH mice. **A** Heatmaps illustrating changes in the expression of hepatic senescence-associated candidate genes in GAN DIO-NASH mice fed the GAN diet for 38-78 weeks as compared to chow-fed mice (*n* = 11-15 per group). **B** Heatmaps illustrating changes in the expression of hepatic senescence-associated candidate genes in GAN DIO-NASH mice receiving dietary intervention (chow reversal) as compared to the corresponding vehicle GAN DIO-NASH mouse group. Mice were fed the GAN diet for 34 weeks before switching to chow feeding (chow-reversal) for 8, 16 or 24 weeks (*n* = 14–16 per group). Chow-fed C57BL/6J mice (Chow vehicle) served as normal controls (*n* = 10). All mice received vehicle (SC, QD, 5 ml/kg) during the intervention phase of the study. Color gradients in heatmaps indicate significantly upregulated (red color) or downregulated (blue color) gene expression (log2-fold change, false discovery rate *p* < 0.05). Unregulated genes are indicated without color fill (white color)

To gain broader insight into the regulation of hepatocellular senescence markers in the model, we initially probed for standard molecular markers of cellular senescence using liver RNAseq data from a recently reported longitudinal study in GAN DIO-NASH (38-72 weeks of GAN diet feeding) [31]. Almost all candidate genes markers of cellular senescence were upregulated, being highly manifest after 38 weeks of GAN diet feeding and remained increased thereafter (Fig. 8A), including molecular markers of the senescence-associated secretory phenotype (SASP, e.g. *Igfbp7, Il1a, Il1b, Mmp12, Mmp13, Plaur, Serpine1* and *Tgfbp1*), cell cycle arrest (e.g. *Bcl2a1a, Bcl2a1b, Bcl2a1d, Cdkn1a, Cdkn2a, Mki67* and various associated transcription factors), and DNA damage (e.g. *Cgas, Chek1, Chek2, Mdc1,* and *Trp53bp1*). A similar hepatic gene expression signature was observed in the current study, which included control mice fed the GAN diet for a total of 42-58 weeks (data not shown). Compared to corresponding GAN DIO-NASH controls, dietary intervention for 8, 16 and 24 weeks promoted highly consistent and relatively similar robust reductions in almost all candidate genes expression markers of SASP, cell cycle arrest and DNA damage (Fig. 8B).

## Discussion

We report that dietary intervention (chow reversal) promotes substantial improvements in metabolic, histological and transcriptional hallmarks of NASH and fibrosis in GAN DIO-NASH mice, a clinical translational model of NASH [26, 27, 31]. Notably, benefits of dietary intervention in GAN DIO-NASH mice were paralleled by robust and sustained suppression of molecular programs linked to cellular senescence, implicating hepatocellular senescence as an important factor contributing to the onset and progression NASH. Collectively, our findings support the concept that targeting mechanisms of hepatocellular senescence may hold potential as a therapeutic approach in NASH.

Cellular senescence refers to a state of irreversible cell-cycle arrest combined with mitochondrial dysfunction and secretion of several proinflammatory cytokines, growth factors and proteases, commonly referred to as SASP [8, 9, 14]. Emerging evidence has linked dysregulation of cellular senescence mechanisms in fibrotic liver diseases, including NASH [32–34]. Increased hepatic senescent cell burden has been reported in NAFLD/NASH patients and preclinical models of NAFLD/NASH [9, 10, 13, 14, 35, 36]. Hepatic accumulation of senescent hepatocytes correlates with NASH severity and can increase to 50-100% in end-stage liver disease [37]. The implications of hepatocellular senescence in the pathogenesis of NASH prompted us to investigate histological and molecular features of cellular senescence in GAN DIO-NASH mice. Consistent with previous reports [26, 27, 31, 38–40], GAN DIO-NASH mice consistently demonstrated metabolic, biochemical and histological hallmarks of progressive NASH and fibrosis. Interestingly, GAN DIO-NASH mice displayed impaired mitochondrial function, reflected by a decreased mitochondrial respiratory and electron transfer system capacity, indicating reduced ability of hepatic mitochondria to adapt to increased metabolic demands as result of progressive hepatocyte fat accumulation [41]. Previous studies have reported increased mitochondrial oxidative function in NAFLD in both humans [42, 43] and animal models [44–46], suggesting liver mitochondria can compensate in early NAFLD. In contrast, NASH patients have been reported to demonstrate decreased liver mitochondrial respiratory activity [43, 47], which has recently been reproduced in AMLN DIO-NASH mice [48], a translational model closely related to the GAN DIO-NASH mouse [26], which implies that mitochondrial adaptive mechanisms become exhausted as the disease progresses. In the current study, impaired mitochondrial lipid metabolism was also suggested by reduced β-HAD activity in GAN DIO-NASH mice, a mitochondrial enzyme marker of medium/short-chain fatty acid oxidative capacity [49], pointing towards decreased fatty acid β-oxidative capacity contributing to defective liver lipid handling in the model. In support of dysregulated fatty acid metabolic flux, a subset of hepatic acyl-CoA thioesterase (*Acot*) genes, being involved in mitochondrial and peroxisomal fatty acid oxidation [50, 51], were differentially regulated in GAN DIO-NASH mice. It is noteworthy that reduced mitochondrial respiration in GAN DIO-NASH mice was temporally closely reflected by overexpression of an extensive number of molecular markers linked to hepatocellular senescence. Given the strong association between mitochondrial function and cellular senescence, we subsequently assessed the hepatic expression of cyclin-dependent kinase inhibitor p21 representing a major target of p53 activity and thus a key marker of DNA damage and cell cycle arrest which are hallmarks of cellular senescence [52]. Consistent with the proportion of senescent hepatocytes being very low in the normal liver [37], p21 expressing hepatocytes were almost absent in chow-fed mice. In contrast, GAN DIO-NASH demonstrated up to 40-fold increase in p21-positive hepatocytes. Stimulated hepatocyte p21 expression in GAN DIO-NASH mice was confirmed by overexpression of the p21-encoding gene (*Cdkn1a*), along with several other CDKI transcripts, including the established cell senescence marker p16 (*Cdkn2a*). In further support of hepatocellular senescence in GAN DIO-NASH mice, we observed robust upregulation of ϒ-H2AX protein and senescence-associated candidate genes reflecting various signaling programs triggering cellular senescence such as DNA damage, cell cycle arrest and SASP. Most extensive perturbations were observed for SASP-associated signaling markers of which the vast majority were highly upregulated, suggesting the acquisition of SASP-defined hepatocytes in GAN DIO-NASH mice. SASP consists of various signalling factors, including cytokines, chemokines, growth modulators, angiogenic factors, proteases, bioactive lipids, extracellular matrix components and matrix metalloproteinases [53]. SASP-defined senescent cells can contribute to chronic inflammation and induce senescence in neighbouring cells [53]. Notably, hepatic SASP has been demonstrated to induce activation of macrophages and drive hepatocellular carcinoma (HCC) progression [54]. Also, GAN DIO-NASH mice demonstrated upregulation of BCl2-family genes, all of which are related to apoptosis resistance and important for the survival of senescent cells [52, 55]. Overall, signatures of mitochondrial deficits and hepatocellular senescence were highly manifest and sustained after ≥ 38 weeks of GAN feeding, making it conceivable that these features characteristic of hepatocellular senescence could be present at earlier disease stages in GAN DIO-NASH mice. Considering that GAN DIO-NASH mice consistently develop HCC after approximately 58 weeks of GAN diet feeding [31], we cannot exclude that ensuing tumorigenesis activity in the liver tissue microenvironment could contribute to shape the molecular signature of hepatocellular senescence in GAN DIO-NASH mice. For example, several cyclins were upregulated (including cyclin-dependent kinases and *Mki67*), which could be interpreted as a cell proliferative signal. Because GAN DIO-NASH mice presented with marked lobular inflammation and fibrosis, upregulation of cyclin expression may also relate to immune and stellate cell proliferative activity in the model.

The role of cellular senescence in NAFLD/NASH is a relatively new area of investigation, and the exact mechanisms by which hepatocellular senescence could drive or, alternatively, be a consequence of NAFLD/NASH remain insufficiently understood. NAFLD patients have been reported to show shortened telomere length and increased expression of markers of cell cycle arrest/DNA damage, including p53, 53BPI, p21 and γH2AX [35, 56–58]. Notably, hepatic senescent liver cells massively accumulate in cirrhotic patients [58]. While senescent hepatocytes can activate hepatic stellate cells (HSCs) by SASP signaling programs [59], spreading of cellular senescence from hepatocytes to HSCs may potentially inactivate HSCs, leading to reduced pro-fibrogenic activity [60]. Preclinical studies in DIO-NAFLD mouse and nutrient-deficient dietary model of NASH models have indicated that steatosis can trigger hepatocyte senescence [61–65]. Conversely, elimination of hepatic senescent cells has been demonstrated to partially reverse steatosis in DIO-NAFLD mice, thus interventions that can prevent or reduce hepatocellular senescence may have therapeutic relevance in the management of NAFLD/NASH [14].

In the absence of approved drug therapies, lifestyle intervention remains the first-line of treatment for NASH [24]. Weight loss following intensive dietary intervention is directly correlated with improved liver histological outcomes in NASH patients [66]. In agreement, a recent study in GAN DIO-NASH mice have indicated improved metabolic and histological hallmarks of NASH after 12 weeks of dietary intervention (chow reversal) [31]. Here, we profiled the therapeutic effect of dietary intervention (chow reversal for 8-24 weeks) in GAN DIO-NASH mice with special emphasis on the impact on markers of hepatocellular senescence. Dietary intervention promoted a robust and sustained weight loss. GAN DIO-NASH mice exhibit significant adiposity, as determined by whole-body MRI [27]. Although we did not determine adipose mass in the current study, weight loss attained by dietary intervention is most likely attributed to reduced fat mass, as previously reported for caloric restriction in GAN DIO-NASH mice [67]. The robust weight loss GAN DIO-NASH mice was paralleled by robust improvements in metabolic markers, NAS and fibrosis histomorphometry (PSR, Col1a1), but not fibrosis stage even after 24 weeks of dietary intervention. It is noteworthy that therapeutic benefits were to a large degree achieved after only 8 weeks of dietary intervention in GAN DIO-NASH mice. As quantitative histological markers of fibrosis were only consistently reduced after 24 weeks of dietary intervention, it may be speculated that even very long dietary intervention periods may preferably reduce de novo collagen synthesis rather than stimulate clearance of pre-existing fibers. This notion is supported by the robust and sustained suppression of α-SMA expression after 8 weeks of dietary intervention, a reliable marker of HSC activation which precedes collagen deposition [68]. To the best of our knowledge, the current study represents the longest duration of dietary intervention applied in a DIO-NASH mouse model, hence also recapitulating the inherent limitations of dietary intervention regimens in the clinical management of NASH.

Enhanced fatty acid oxidation to increase lipid disposal and reducing lipotoxicity by increasing mitochondrial content and function has been proposed having therapeutic potential in NASH [69, 70]. Interestingly, beneficial metabolic and histological outcomes of dietary intervention were paralleled by significant improvements in mitochondrial respiration and fatty acid oxidative capacity, implicating improved mitochondrial health as an important mechanism underlying complete reversal of severe steatosis in GAN DIO-NASH mice following dietary intervention. Bariatric surgery-induced weight loss improves adipose tissue and liver mitochondrial respiration in NAFLD/NASH patients, being predominantly ascribed to increased mitochondrial content [71]. Although mitochondrial density was not specially assessed in the current study, citrate synthase activity, a surrogate marker for mitochondrial density [72], was unaltered following long-term (≥ 16 weeks) dietary intervention, rendering it less likely that dietary intervention led to increased hepatic mitochondrial content in GAN DIO-NASH mice. Collectively, dietary intervention corrected mitochondrial respiratory deficits concurrent with widespread suppression of hepatocellular senescence molecular signatures, tentatively linking improved liver health in GAN DIO-NASH mice to clearance of senescent hepatocytes or, alternatively, inhibition of hepatocellular senescence signaling mechanisms.

## Conclusions

The GAN DIO-NASH mouse model demonstrates impaired hepatic mitochondrial function and molecular signatures of hepatocellular senescence, as also reported in human NASH, lending further support to clinical translatability of the model. Notably, dietary intervention (chow reversal) led to substantial improvements in hallmarks of NASH concurrent with complete reversal of mitochondrial respiratory deficits and suppression of hepatocellular senescence. In conclusion, our data supports therapeutic potential in targeting mechanisms of hepatocellular senescence in the management of NASH, and further highlights the utility of the GAN DIO-NASH mouse model for the evaluation of drugs with potential senolytic therapeutic properties in NASH.

## Data Availability

The RNA sequencing datasets generated in the current study are available in the Gene Expression Omnibus (GEO) repository [https://www.ncbi.nlm.nih.gov/geo/; accession number GSE246328].

## Abbreviations

ALT: Alanine aminotransferase
AST: Aspartate aminotransferase
α-SMA: Alpha-smooth muscle actin
β-HAD: Beta-hydroxyacyl-CoA dehydrogenase
CRN: NASH Clinical Research Network
Col1a1: Collagen-1a1
DIO: Diet-induced obese
ETS: Electron transport system
FCCP: Carbonyl cyanide p-trifluoro-methoxyphenyl hydrazine
GAN: Gubra-Amylin NASH
HCC: Hepatocellular carcinoma
GHOST: Gubra Histopathological Objective Scoring Technology
HE: Hematoxylin-eosin
HSC: Hepatic stellate cell
IP10: Interferon gamma-induced protein 10
MCP-1: Monocyte chemoattractant protein-1
MRC: Mitochondrial respiratory chain
NAFLD: Non-alcoholic fatty liver disease
NASH: Non-alcoholic steatohepatitis
NAS: NAFLD Activity Score
PCA: Principal component analysis
PSR: Picro-Sirius Red
ROS: Reactive oxygen species
SASP: Senescence-associated secretory phenotype
TC: Total cholesterol
TG: Triglycerides
TNF-α: Tumor necrosis factor alpha
